# Preoperative predictors of early patient-reported recovery trajectories after major surgery: a secondary analysis of a randomized controlled trial

**DOI:** 10.1016/j.sopen.2026.10.002

**Published:** 2026-10-19

**Authors:** Miriam van der Velde, Jessica M. de Graaf, Edwin Geleijn, Karin Valkenet, Marike van der Leeden

**Affiliations:** a Research Group Innovation of Human Movement Care, Center for Healthy and Sustainable Living, HU University of Applied Sciences, Heidelberglaan 7, Utrecht, 3584 CS, the Netherlands; b Department of Rehabilitation, Physical Therapy Science and Sports, University Medical Center Utrecht, Utrecht University, Heidelberglaan 100, Utrecht, 3584 CX, the Netherlands; c Department of Rehabilitation Medicine, Amsterdam University Medical Center, Vrije Universiteit Amsterdam, De Boelelaan 1117, Amsterdam, 1081 HV, the Netherlands; d Amsterdam Movement Sciences, Ageing and Vitality, De Boelelaan 1117, Amsterdam, 1081 HV, the Netherlands

**Keywords:** Major surgery, Postoperative recovery, PROMIS, Physical functioning, Social participation

## Abstract

**Objective:**

To examine recovery trajectories of Physical Functioning (PF) and the Ability to Participate in Social Roles and Activities (APS) after major surgery, and identify preoperative predictors.

**Background:**

Recovery after major surgery is typically evaluated at isolated time points, limiting insight into recovery dynamics. Understanding recovery trajectories and their determinants may inform prognostic model development.

**Methods:**

This exploratory secondary analysis used data from a multicenter randomized controlled trial including adults undergoing major elective surgery. Patient-Reported Outcomes Measurement Information System (PROMIS) Computer Adaptive Test PF and APS were assessed preoperatively and at 1, 3, 6, and 12 weeks post discharge. Recovery trajectories were modeled using linear mixed effects models with random intercepts and slopes. Preoperative characteristics were examined using interaction terms for time and time^2^.

**Results:**

Among 369 patients, PF and APS followed curvilinear patterns. For PF, older age (β = −0.04, 95% CI [−0.06 to −0.02]), unintentional weight loss and higher comorbidity were associated with slower recovery. Higher baseline PF predicted with faster recovery. Differences exceeding the Minimal Important Change (MIC) were observed for age and baseline PF.

For APS, older age was also associated with slower recovery (β = −0.04, 95% CI [−0.08 to −0.004]), and intermediate education (vs low) with differences in recovery curvature. Higher baseline APS predicted higher postoperative scores. MIC-exceeding differences were observed for baseline APS and age.

**Conclusions:**

Early postoperative recovery is curvilinear. Multiple preoperative factors were associated with recovery trajectories, supporting their role as candidate predictors for future trajectory-based prognostic models.

**Trial registration:**

This study was approved by the Medical Ethical Committee of Amsterdam University Medical Center, location VUmc, under registration number NL61503.029.18.

## Introduction

Recovery from major surgery is a complex and multidimensional process [1]. The body's stress response to surgery, along with the effects of anesthesia, perioperative immobility and possible complications, can lead to delayed or poor recovery [2]. These problems often extend beyond the hospital stay, affecting patients' quality of life and placing a considerable burden on the healthcare system and society, due to prolonged hospital stays, readmissions, increased healthcare utilization, and delayed return to normal functioning [3], [4].

Traditionally, studies evaluating postoperative recovery in patients who underwent major surgery evaluate medical outcomes, such as complications, length of stay or readmissions. However, from the patient's perspective, the ability to perform physical and social activities as they could prior to surgery, is the most important aspect of recovery [5], [6]. Also, the growing emphasis on value-based healthcare has highlighted the importance of patient-reported outcome measures (PROMs) in evaluating postoperative recovery over time [7], [8].

Recovery after major surgery typically follows a course marked by an initial decline, followed by a gradual return to baseline levels [6]. However, recovery from major surgery is a highly individual and variable process. Some patients recover fast and quickly return to their normal activities, while others face prolonged activity limitations [9], [10], [11], [12], [13]. In a study of patients undergoing gastrointestinal or bladder oncological surgery, 49% had not recovered to their baseline level of functioning three months after surgery [11]. Similarly, in a study of patients with lung cancer, 76% had not recovered to their preoperative level of physical functioning at three months postoperatively [13]. Additionally, a study examining the patterns of recovery following abdominal surgery found several distinct clusters of recovery patterns [12].

These findings underscore the need to understand which preoperative factors are associated with how patients recover over time, rather than at isolated time points.

Recovery after major surgery is shaped by a combination of preoperative, intraoperative, and postoperative factors. Among these, preoperative characteristics, such as age, comorbidities and risk behaviors like physical inactivity and smoking, are particularly valuable, as they are known prior to surgery. This provides a critical window of opportunity to identify patients at risk of delayed or poor recovery and to implement targeted interventions, such as prehabilitation [14], [15].

Therefore, this secondary analysis of data from a randomized controlled trial aims to gain insight in the recovery trajectories of physical functioning and social participation among patients who underwent major surgery and explore which preoperative characteristics are associated with those trajectories.

## Methods

### Study design

This study is a secondary analysis of a single-blind parallel multicenter randomized controlled trial evaluating the effectiveness of the Be Prepared mHealth app when added to usual care, in a population of patients undergoing major elective surgery [16]. The Be Prepared app is a smartphone app with pre- and postoperative information and instructions on changing risk behavior prior to major elective surgery. The primary outcome was postoperative physical functioning (PF) up to 12 weeks after hospital discharge. Secondary outcomes included post-operative social participation, self-reported recovery, health-related quality of life, postoperative outcomes, and patient satisfaction. Patients were recruited at three university hospitals and one general hospital in the Netherlands between June 2020 and June 2022. Results of the RCT showed that the use of the Be Prepared app led to a small statistically significant but not clinically relevant improvement in the postoperative recovery of PF from 1 week to 12 weeks after hospital discharge, compared to patients receiving usual care. No significant differences were found for social participation [16]. The patients included in the parent trial were treated as a single cohort of patients undergoing major surgery and group allocation was included in the analysis to account for potential intervention effects. This study was approved by the Medical Ethical Committee of Amsterdam University Medical Center, location VUmc, under registration number NL61503.029.18.

### Participants

Patients were recruited at the pre-operative assessment at the participating hospitals. Prior to participation, informed consent was obtained from all patients, and eligibility was checked. Patients were eligible for participation if they were scheduled for major elective surgery, defined as procedures with an expected postoperative hospital stay of at least 2 nights, across multiple surgical specialties (including gastrointestinal, cardiothoracic, urological/gynaecological, and others). Patients had to be aged 18 years or older and exhibited one or more risk behaviors (i.e., smoking, drinking, low physical activity, low muscle-strengthening activity, unintentional weight loss). Exclusion criteria included insufficient comprehension of the Dutch language, lack of access to a mobile device, scheduled brain surgery, surgery planned within seven days, participation in conflicting studies, or enrollment in an intensive preoperative care program (e.g., an exercise regimen) [16].

### Outcomes

All outcomes were collected within the parent RCT using online questionnaires and electronic health records; no additional data collection was performed for this secondary analysis. Questionnaires were administered at five timepoints: prior to surgery (T0), one week (T1), three weeks (T2), six weeks (T3) and twelve weeks (T4) after hospital discharge.

Recovery of physical functioning and social participation were assessed at all timepoints. Physical functioning was measured using the Dutch version of the Patient-Reported Outcomes Measurement Information System (PROMIS) – Physical Functioning (PF) Computer Adaptive Test (CAT). This computer-administered tool selects 4–12 questions (5-point scale) from a 121-item bank, based on a participants previous answers. The items assess daily physical activities, with higher scores indicating better functioning. Standard PROMIS CAT stopping rules were applied [17]. Social participation was measured using the Dutch version of the CAT PROMIS Ability to Perform Social roles and activities (APS). Patients received 4–12 items from a 35-item bank, rated on a 5-point scale, assessing their ability to engage in social interactions. Higher scores indicated greater participation in social activities [18].

Preoperative characteristics, collected prior to surgery (T0) within the RCT, are used in this secondary analysis to explore factors associated with postoperative recovery trajectories. These characteristics were selected a priori based on clinical expertise, existing literature and availability within the parent trial. Preoperative physical functioning and social participation were obtained using the Dutch versions of the CAT-PROMIS as described above. Age, gender, body mass index (BMI), American Society of Anesthesiologist (ASA) classification, Charlson Comorbidity Index (CCI) and type of surgery were extracted from electronic health records. ASA classification was grouped into two categories: levels I–II and levels III–IV. The CCI was categorized into three levels of comorbidity: mild (score 0–1), moderate (score 2), and severe (score ≥ 3). Type of surgery was classified as oncological or non-oncological. Level of education was obtained via an online questionnaire and categorized as low, intermediate or high.

Risk behaviors were assessed using a self-report questionnaire, with each behavior (smoking, consumption of seven or more alcoholic beverages per week, engaging in less than 30 min of moderate-intensity physical activity per day, performing muscle-strengthening activities on fewer than two days per week, and experiencing unintentional weight loss of more than 3 kg in the past month) scored dichotomously as present or absent [16]. In addition, a cumulative risk behavior burden (range 1–5) was calculated to capture the number of co-occurring behaviors and categorized as low (1–2 risk behaviors) and high (3–5 risk behaviors).

### Statistical analyses

All analyses were conducted in R version 4.4.1 (R foundation for statistical Computing, Vienna, Austria). The R package lme4 was used to fit linear mixed-effects models to explore postoperative recovery trajectories and to examine the influence of preoperative characteristics [19], [20], [21]. The two outcomes, physical functioning (PROMIS-PF) and social participation (PROMIS-APS) were analyzed separately. In all models, time (post-surgery) was treated as a continuous variable to model recovery over time. Random intercepts and slopes were included to account for individual variation. Group allocation (intervention versus control) was included as a fixed effect to control for group differences. Missing data were handled through maximum likelihood estimation.

To establish the functional form of recovery, we compared models with linear and quadratic terms for time (time^2^). These model comparisons were performed separately for PROMIS-PF and PROMIS-APS. Both models included group allocation as a fixed effect and random intercepts and slopes for participants. Model fit was evaluated using the Akaike Information Criterion (AIC), the Bayesian Information Criterion (BIC), and likelihood-ratio tests (ANOVA). Lower AIC and BIC values indicated a better model fit as well as a significant likelihood-ratio test [19]. The model with the best fit for each outcome according to these criteria was selected as the base model for subsequent predictor analyses.

The aim of this study was to explore associations between individual preoperative characteristics and postoperative recovery trajectories rather than to develop a multivariable prediction model. To identify preoperative predictors of recovery, a separate univariate mixed-effects model was fitted for each preoperative characteristic. First we added each preoperative characteristic as a fixed main effect to the outcome specific base model. Subsequently, characteristic-by-time interaction terms (time and time^2^) were added to evaluate whether the association between the characteristic and the outcome changed over the postoperative recovery period. Models including only the main effect were compared with models additionally including the interaction terms using AIC, BIC and likelihood-ratio tests. If adding interactions with time and time^2^ improved model fit, the preoperative characteristic was considered to be associated with recovery over time. For categorical predictors, the reference category was specified a priori and the remaining categories were included using dummy coding. Consequently, regression coefficients reflected comparisons between each category and the reference category. Overall associations of categorical predictors with recovery trajectories were evaluated based on model comparisons.

Only for characteristics for which the interaction model showed a better fit than the main-effects model, we interpreted the time-dependent coefficients (time and time^2^ interactions). A significant interaction between time and the preoperative characteristic represented differences in the linear recovery over time, whereas a significant interaction between time^2^ and the preoperative characteristic captured differences in the curvature of recovery trajectories. For these characteristics, model-based predicted recovery trajectories were visualized to facilitate interpretation of the combined linear and quadratic time effects.

Statistical significance was determined using an alpha level of 0.05. All mixed-effects models were estimated using maximum likelihood to ensure valid comparisons between models differing in fixed effects. Minimal important changes (MICs) were calculated to provide a clinically oriented reference point for interpreting observed differences in postoperative recovery. MICs were estimated using a distribution-based approach [22]. The MIC was defined as 0.5 times the standard deviation of individual change scores from preoperative to 12 weeks post-surgery. The resulting MIC thresholds were 3.79 for PROMIS-PF and 7.50 for PROMIS-APS. In this study the MIC served solely as a reference point to help interpret the size of the predicted recovery differences over time. It was not used as a criterion to determine the clinical relevance of between-group effects [22], [23].

## Results

### Study population and patient characteristics

A total of 472 patients were enrolled, of whom 369 (78%) had at least one follow-up measurement and were included in the final analysis.

Patients had a mean age of 62.0 (SD = 11.9), 60.4% were men, and 41.5% had a high level of education. The mean preoperative PROMIS-PF was 45.3 (SD = 8.3) and the mean PROMIS-APS score prior to surgery was 50.2 (SD = 11.0). Preoperative patient characteristics are shown in Table 1.

**Table 1:** Preoperative characteristics of participants.

Characteristics	*n* = 369
Age (years), mean (SD)	62.0 (11.9)
Men, n (%)	223 (60.4)
BMI^a^ (kg/m^2^), mean (SD)	26.5 (5.2)
Surgical oncology, n (%)	200 (54.2)
Surgical specialty, n (%)	
Gastrointestinal	124 (33.5)
Cardiothoracic	103 (27.9)
Urology and gynaecology	49 (13.3)
Orthopaedic and Spine	34 (9.2)
Head and Neck	30 (8.1)
Vascular	19 (5.1)
Plastic	8 (2.2)
Neurosurgical	2 (0.5)
ASA^b^ classification, n (%)	
I & II	208 (56.4)
III & IV	144 (39)
Missing	17 (4.6)
Risk behaviors^c^, n (%)	
Smoking	32 (8.7)
Alcohol consumption	32 (8.7)
Physical inactivity	289 (78.3)
No muscle strengthening activities	346 (93.8)
Unintentional weight loss	49 (13.3)
Risk behavior burden, n (%)	
Low (1 or 2 risk behaviors)	317 (85.9)
High (3, 4 or 5 risk behaviors)	52 (14.1)
Level of education^d^, n (%)	
Low	96 (26.0)
Intermediate	113 (30.6)
High	153 (41.5)
Missing	7 (1.9)
CCI^e^, n (%)	
None	85 (23.0)
Mild (1–2)	189 (51.2)
Moderate (3–4)	59 (16.0)
Severe (≥ 5)	33 (8.9)
Preoperative CAT PROMIS-PF^f^ score, mean (SD)	45.3 (8.3)
Preoperative CAT PROMIS-APS^g^ score, mean (SD)	50.2 (11.0)
Intervention group, n (%)	188 (50.9)

a BMI: body mass index.

b ASA: American Society of Anesthesiologists.

c Multiple response options.

d Low: preschool, primary school, lower vocational education; intermediate: secondary education, intermediate vocational education; high: higher vocational education, university, postgraduate.

e CCI: Charlson Comorbidity Index.

f CAT PROMIS-PF: Computer Adaptive Test PROMIS Physical Functioning.

g CAT PROMIS-APS: Computer Adaptive Test PROMIS Ability to Perform Social roles and activities.

### Recovery trajectory of physical functioning

Comparison of a linear and quadratic time model showed that the model including a quadratic time term provided a significantly better fit than the linear model (∆ AIC = −137.85, ∆ BIC = −132.72, $\chi^{2}$(1) = 139.85, *p* < .001). The base model therefore included time, time,^2^ group allocation, and random intercepts and slopes.

### Preoperative predictors of recovery trajectories of physical functioning

Likelihood ratio tests, supported by lower AIC values (∆ AIC = 2.73–29.09) and mostly small changes in BIC (∆ BIC = 4.84 – −22.45) indicated that including interactions with time and time^2^ significantly improved model fit for age, surgical oncology, alcohol consumption, unintentional weight loss, risk behavior burden, level of education, comorbidity, and preoperative physical functioning ($\chi^{2}$ = 7.18–41.09, df = 2–6, *p* < .05; Table 2). The estimated effects of the predictors associated with recovery over time, including strength and direction over time, are presented in Table 3. For categorical predictors, the *p*-values reported in Table 2 represent overall likelihood-ratio tests of the predictor, whereas the *p*-values reported in Table 3 represent individual category contrasts and interaction terms relative to the prespecified reference category.

**Table 2:** Model comparisons for preoperative predictors of recovery trajectories of physical functioning.

Baseline characteristic	∆ AIC	∆ BIC	$\chi^{2}$ (df)	*P*-value
**Age**	**15.10**	**4.84**	**19.10 (2)**	**<0.001**
Gender	1.06	−9.20	5.06 (2)	0.080
BMI^a^	−2.59	−12.86	1.41 (2)	0.495
ASA^b^ classification	−3.75	−13.91	0.26 (2)	0.880
**Surgical oncology**	**9.53**	**−0.74**	**13.53 (2)**	**0.001**
Physical inactivity	−3.04	−13.31	0.96 (2)	0.620
No muscle strengthening activities	−1.24	−11.50	2.76 (2)	0.251
**Alcohol consumption**	**10.21**	**−0.05**	**14.21 (2)**	**0.001**
**Unintentional weight loss**	**2.73**	**−7.53**	**6.73 (2)**	**0.035**
Smoking	0.05	−10.22	4.05 (2)	0.132
**Risk behavior burden**	**7.10**	**−3.17**	**11.10 (2)**	**0.004**
**Level of education**	**8.33**	**−22.45**	**20.33 (6)**	**0.002**
**CCI**^c^	**29.09**	**−1.64**	**41.09 (6)**	**<0.001**
**Preoperative CAT PROMIS-PF**^d^**score**	**3.18**	**−7.08**	**7.18 (2)**	**0.028**

ΔAIC and ΔBIC indicate changes in model fit relative to the base model. χ^2^ (df) and *p*-values are derived from likelihood ratio tests. Bold signifies significant *p*-values.

a BMI: body mass index.

b ASA: American Society of Anesthesiologists.

c CCI: Charlson Comorbidity Index.

d CAT PROMIS-PF: Computer Adaptive Test PROMIS Physical Functioning.

**Table 3:** Fixed-effect estimates of preoperative predictors of recovery trajectories of physical functioning.

Baseline characteristic	β (95% CI)	*P*-value
Age	−0.02 (−0.10 to 0.07)	0.733
**Age:time**	**−0.04 (−0.06 to -0.02)**	**0.001**
**Age:time**^**2**^	**0.002 (0.0003 to 0.0036)**	**0.024**
Surgical oncology	2.57 (0.49 to 4.65)	0.016
**Surgical oncology:time**	**−0.83 (−1.37 to -0.30)**	**0.002**
**Surgical oncology:time**^**2**^	**0.043 (0.006 to 0.081)**	**0.024**
Alcohol consumption	6.17 (2.54 to 9.81	0.001
**Alcohol consumption:time**	**−1.14 (−2.08 to -0.20)**	**0.018**
Alcohol consumption:time^2^	0.044 (−0.023 to 0.110)	0.194
Unintentional weight loss	2.37 (−0.63 to 5.36)	0.121
**Unintentional weight loss:time**	**−1.00 (−1.76 to -0.24)**	**0.010**
**Unintentional weight loss:time**^**2**^	**0.069 (0.015 to 0.123)**	**0.012**
Level of education		
Intermediate vs low	−0.87 (−3.66 to 1.92)	0.541
Intermediate vs low:time	0.24 (−0.47 to 0.95)	0.510
Intermediate vs low:time^2^	0.002 (−0.048 to 0.051)	0.951
High vs low	−2.87 (−5.47 to −0.28)	0.030
**High vs low:time**	**1.07 (0.42 to 1.73)**	**0.001**
**High vs low:time**^**2**^	**−0.05 (−0.10 to -0.01)**	**0.029**
CCI^a^		
Mild vs none	1.84 (−0.77 to 4.44)	0.167
Mild vs none:time	−0.36 (−1.03 to 0.31)	0.288
Mild vs none:time^2^	0.012 (−0.035 to 0.060)	0.615
Moderate vs none	5.54 (2.19 to 8.90)	0.001
**Moderate vs none:time**	**−2.35 (−3.21 to -1.48)**	**<0.001**
**Moderate vs none:time**^**2**^	**0.13 (0.07 to 0.19)**	**<0.001**
Severe vs none	2.47 (−1.58 to 6.52)	0.232
**Severe vs none:time**	**−1.18 (−2.19 to -0.17)**	**0.023**
Severe vs none:time^2^	0.06 (−0.01 to 0.13)	0.119
Risk behavior burden (low vs high)	4.12 (1.17 to 7.06)	0.006
**Risk behavior burden:time**	**−1.24 (−1.99 to -0.48)**	**0.001**
**Risk behavior burden:time**^**2**^	**0.07 (0.02 to 0.13)**	**0.006**
Preoperative CAT PROMIS-PF^b^ score	0.42 (0.30 to 0.53)	<0.001
**Preoperative CAT PROMIS-PF score:time**	**0.03 (0.003 to 0.07)**	**0.034**
Preoperative CAT PROMIS-PF score:time^2^	−0.002 (−0.004 to 0.001)	0.131

Regression coefficients, 95% confidence intervals and *p*-values for the main effects and their interactions with time and time^2^ are presented. For categorical predictors, these *p*-values correspond to individual category contrasts relative to the reference category. Bold signifies significant *p*-values for the interactions with time and time^2^.

a CCI: Charlson Comorbidity Index.

b CAT PROMIS-PF: Computer Adaptive Test PROMIS Physical Functioning.

Interactions with time indicate that the rate of postoperative recovery varies as a function of the predictor. Positive interactions indicate a steeper improvement over time at higher values or levels of the predictor relative to reference, whereas negative interactions indicate a less steep improvement over time. Interactions with time^2^ reflect differences in the curvature of recovery trajectories over time as a function of the predictor. A positive interaction with time^2^ suggests a recovery trajectory that is initially more gradual and accelerates at later time points, resulting in a more delayed flattening of the recovery trajectory. Conversely, a negative interaction with time^2^ suggests stronger early improvement followed by earlier attenuation or plateauing of recovery. The interpretation of these interactions depends on the combined effects of the linear and quadratic time terms and is therefore most clearly illustrated through plots of predicted recovery trajectories (Fig. 1). Although the models included a quadratic time term to capture non-linear trends, predicted values were plotted at the observed follow-up time points (T1–T4) and connected linearly for visual clarity.

**Fig. 1:**
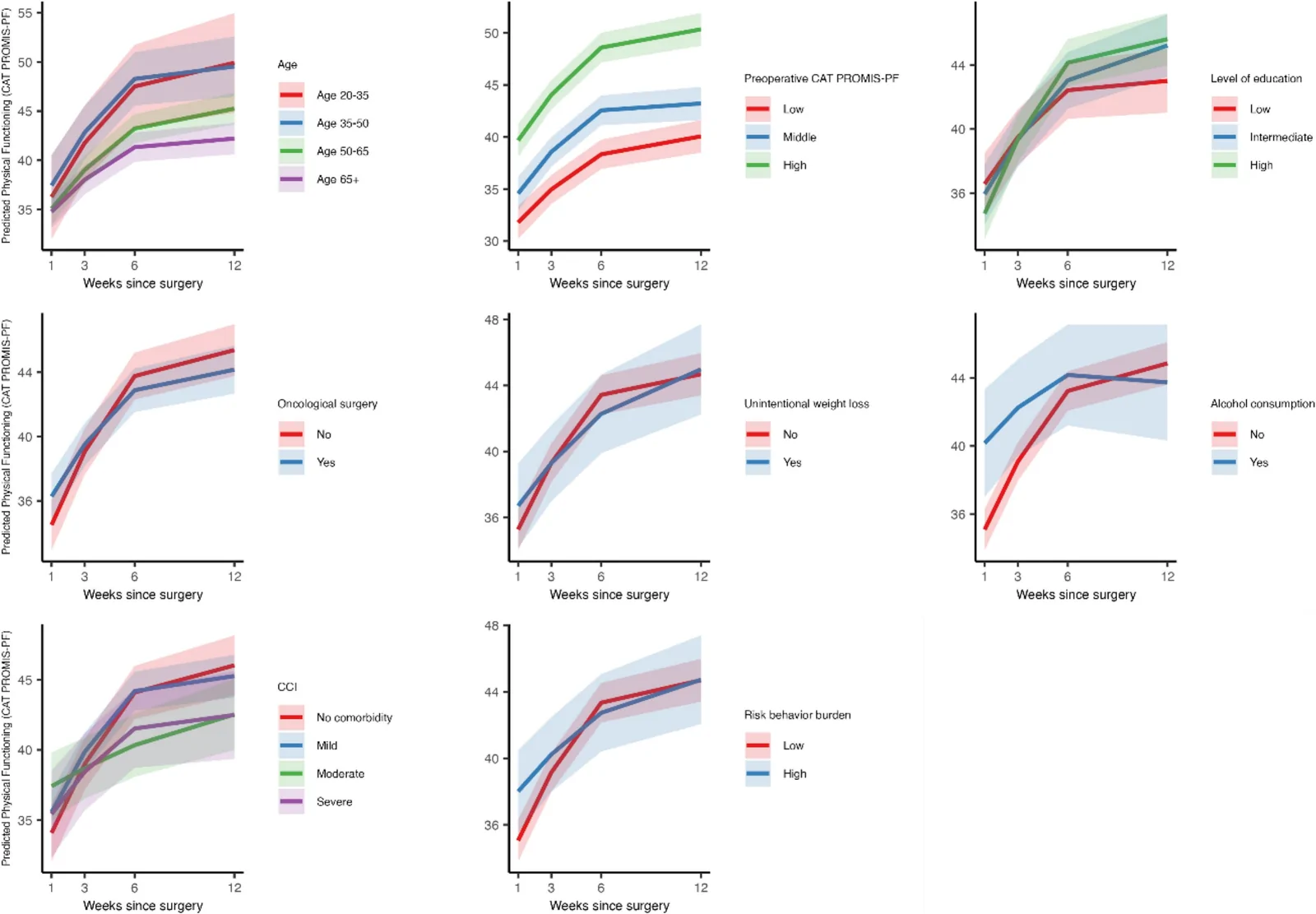
Predicted recovery trajectories of physical functioning over time for each significant predictor. Shaded areas represent 95% confidence intervals around the predicted means. Predicted values are shown at all follow-up time points (T1-T4). The lines between time points are drawn linearly for visual clarity only and do not imply a linear relationship between timepoints. Continuous predictors (age and preoperative physical functioning) were categorized for visualization purposes only. Age was categorized into 15-year intervals and preoperative scores were divided into tertiles based on the sample.

As an illustration, age was associated with differences in both the rate and curvature of postoperative recovery (time: B = −0.04, 95% CI [−0.06, −0.02], *p* = .001; time^2^: B < 0.01, 95% CI [0.0003, 0.0036], *p* = .024, Table 3). As shown in Fig. 1, older patients appeared to recover more slowly in the early postoperative period, but their improvement decelerated less over time compared to younger patients. Conversely, younger patients initially recovered more quickly, but their improvement slowed more strongly as time progressed.

Differences exceeding the MIC of 3.79 were found for preoperative physical functioning and age. No MIC-exceeding differences were observed for surgical oncology, comorbidity, unintentional weight loss, risk behavior burden, education, or alcohol consumption. For all outcomes refer to Table 2, Table 3.

### Recovery trajectory of social participation

Similar to physical functioning, a model including a quadratic time term provided the best fit for social participation, indicating a curvilinear recovery trajectory (∆ AIC = −5.35, ∆ BIC = −0.23, $\chi^{2}$(1) = 7.35, *p* = .007).

### Preoperative predictors of recovery trajectories of social participation

Model comparisons showed that age, surgical oncology, level of education, and preoperative social participation were significantly associated with recovery (*p* < .05 for all, see Table 4). The estimated effects over time are presented in Table 5, and the predicted recovery trajectories are illustrated in Fig. 2.

**Table 4:** Model comparisons for preoperative predictors of social participation recovery trajectories.

Baseline characteristic	Delta AIC	Delta BIC	X2 (df)	*P*-value
Age	**2.94**	**−7.31**	**6.94 (2)**	**0.031**
Gender	−3.72	−13.97	0.28 (2)	0.870
BMI^a^	−2.19	−12.44	1.81 (2)	0.404
ASA classification^b^	−3.47	−13.62	0.53 (2)	0.767
**Surgical oncology**	**4.48**	**−5.76**	**8.48 (2)**	**0.014**
Physical inactivity	−3.72	−11.30	2.95 (2)	0.229
No muscle strengthening activities	−1.05	−13.01	1.23 (2)	0.540
Alcohol consumption	−2.77	−13.18	1.07 (2)	0.586
Unintentional weight loss	−2.93	−11.63	2.62 (2)	0.270
Smoking	−1.38	−10.86	3.39 (2)	0.184
**Risk behavior burden**	**−0.61**	**−6.78**	**7.47 (2)**	**0.024**
**Level of education**	**3.47**	**−23.34**	**19.40 (6)**	**0.004**
CCI^c^	−4.18	−32.54	10.15 (6)	0.118
**Preoperative CAT PROMIS-APS**^d^**score**	**7.40**	**−3.64**	**10.61 (2)**	**0.005**

ΔAIC and ΔBIC indicate changes in model fit relative to the base model. Positive ΔAIC and ΔBIC values reflect a decrease in AIC and BIC for the model including the interactions with time and time^2^, indicating model improvement. χ^2^ (df) and *p*-values are derived from likelihood ratio tests. Bold signifies significant *p*-values.

a BMI: body mass index.

b ASA: American Society of Anesthesiologists.

c CCI: Charlson Comorbidity Index.

d CAT PROMIS-APS: Computer Adaptive Test PROMIS Ability to Perform Social roles and activities.

**Table 5:** Fixed-effect Estimates of Preoperative Predictors of Recovery Trajectories of Social Participation.

Baseline characteristic	β (95% CI)	*P*-value
Age	0.05 (−0.07 to 0.16)	0.455
**Age:time**	**−0.04 (−0.08 to −** **0.004)**	**0.032**
Age:time^2^	0.002 (−0.001 to 0.005)	0.164
Surgical oncology	1.97 (−0.81 to 4.75)	0.165
Surgical oncology:time	−0.78 (−1.64 to 0.08)	0.076
Surgical oncology:time^2^	0.02 (−0.04 to 0.08)	0.446
Level of education		
Intermediate vs low	1.52 (−2.20 to 5.23)	0.423
Intermediate vs low:time	−1.08 (−2.23 to 0.08)	0.068
**Intermediate vs low:time**^**2**^	**0.11 (0.03 to 0.19)**	**0.009**
High vs low	−2.59 (−6.03 to 0.85)	0.139
High vs low:time	0.31 (−0.76 to 1.37)	0.569
High vs low:time^2^	0.03 (−0.04 to 0.10)	0.429
**Preoperative PROMIS-APS score**	**0.26 (0.14 to 0.38)**	**<0.001**
Preoperative PROMIS-APS score:time	0.03 (−0.005 to 0.07)	0.089
Preoperative PROMIS-APS score:time^2^	−0.001 (−0.003 to 0.002)	0.616

Regression coefficients, 95% confidence intervals and *p*-values for the main effects and their interactions with time and time^2^ are presented. For categorical predictors, these *p*-values correspond to individual category contrasts relative to the reference category. Bold signifies significant *p*-values for the interactions with time and time^2^.

**Fig. 2:**
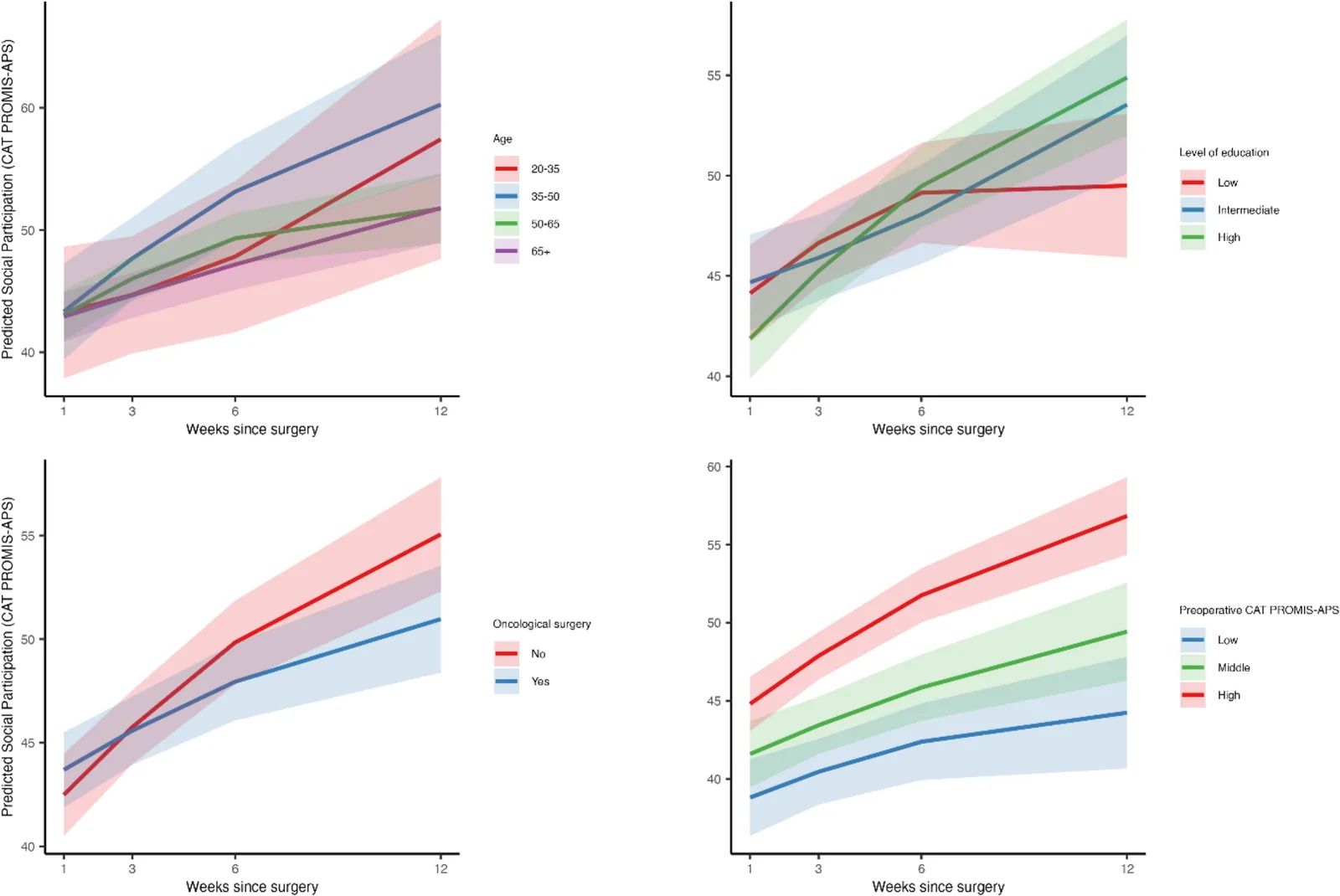
Predicted trajectories of recovery of social participation over time for each significant preoperative characteristic. Shaded areas represent 95% confidence intervals around the predicted means. Predicted values are shown at all follow-up time points (T1-T4). The lines between time points are drawn linearly for visual clarity only and do not imply a linear relationship between timepoints. Continuous predictors (age and preoperative social participation) were categorized for visualization purposes only. Age was categorized into 15-year intervals and preoperative scores were divided into tertiles based on the sample.

Differences exceeding the MIC of 7.50 were observed between patients with lower versus higher preoperative social participation and between older and younger patients, whereas no MIC-exceeding differences were observed for oncologic surgery or education.

## Discussion

In this cohort, postoperative recovery of physical functioning and social participation over 12 weeks followed a curvilinear pattern. This pattern shows that, on average, recovery progresses in a gradually decelerating manner: improvements were evident early in the post-discharge period and tended to flatten over time. However, not all patients undergoing major surgery showed the same recovery trajectory. We found several preoperative characteristics that were statistically associated with the rate and curvature of recovery in physical functioning and social participation. By modelling trajectories rather than isolated time points, our analysis adds nuance to the recovery literature and identifies patterns that are not visible when outcomes are assessed at single follow-up moments.

For physical functioning, patients with older age, surgical oncology, unintentional weight loss, moderate comorbidity, and a higher risk-behavior burden showed a pattern of slower early postoperative recovery combined with less deceleration of recovery over time. Alcohol consumption and severe comorbidity were associated with slower early postoperative recovery but did not significantly influence the curvature of the recovery trajectory, indicating more uniformly reduced postoperative improvement rather than a distinct pattern over time. Patients with a high level of education (compared with those with a low level of education) and better preoperative physical functioning showed faster early improvement, with highe education additionally demonstrating earlier deceleration, suggesting that these individuals may recover more rapidly after surgery before reaching a faster plateau. Differences exceeding the MIC were found only for age and preoperative PF. This underscores that age and preoperative PF may have clinical relevance at the individual level. Other statistically significant factors did not exceed the MIC. However, these factors help to characterize between-patient variability in recovery trajectories and may inform the development of future multivariable prognostic models.

For social participation, a limited number of preoperative characteristics showed significant associations with postoperative recovery trajectories. Older age was associated with slower early postoperative improvement but did not significantly influence the curvature of the recovery trajectory. Patients with an intermediate education level showed a steeper curvature of recovery compared to those with low education, suggesting modest early gains that accelerate later in follow-up. Higher preoperative social participation was associated with higher postoperative levels across all time points, yet it did not modify the rate or curvature of the recovery trajectory, indicating a stable advantage throughout recovery rather than a distinct temporal pattern. Although model comparisons hinted that surgical oncology and risk behavior burden might influence recovery trajectories, individual slope and curvature terms were not significant, and these factors should therefore be interpreted with caution. Differences exceeding the MIC were observed between lower vs higher preoperative social participation and between older vs younger patients.

These factors are broadly consistent with prior studies in different major surgery populations that assessed recovery at specific postoperative time points and across diverse recovery outcomes. For example, older adults frequently experience incomplete recovery after major surgery, which may reflect age related declines in physiological reserve and resilience [24], [25], [26]. Additionally, surgical oncology may contribute to slower recovery because cancer surgeries typically involve greater surgical stress responses and cancer patients may also suffer from the effects of (neo)adjuvant treatment, which may further influence the quality of recovery [27]. The presence of multiple (chronic) conditions and a higher burden of unhealthy preoperative behaviors have also been shown to impair recovery [28]. Patients with comorbidities and multiple risk behaviors may enter surgery in a less physically fit state, making functional recovery after surgery slower and less efficient [29], [30]. Our findings extend to this evidence by showing how these factors affect both the rate and curvature of recovery, offering a more comprehensive understanding of postoperative recovery.

We evaluated the same candidate predictors for both outcomes. A larger number of preoperative characteristics were statistically associated with PROMIS-PF than with PROMIS-APS. This likely reflects the more direct relationship between preoperative physical and clinical characteristics and physical functioning, whereas social participation is influenced by additional psychosocial and environmental factors that were not captured in our candidate predictors. These findings suggest that physical recovery may be more predictable from preoperative characteristics, whereas social participation may require consideration of broader psychosocial and contextual determinants, not measured in our dataset [18].

An interesting observation in this exploratory analysis is that several factors traditionally associated with increased postoperative morbidity and mortality, such as ASA classification and BMI, did not emerge as significant predictors of postoperative physical functioning or social participation in our models. These findings highlight an important distinction between clinical risk and patient-reported recovery. Predictors of morbidity and mortality do not necessarily translate into predictors of how patients perceive and experience their physical and social recovery in the weeks after discharge. Patient-reported outcomes after surgery appear to be more strongly influenced by baseline functional status, psychosocial factors and behavioral aspects rather than by traditional perioperative risk indicators alone [28], [31]. These insights argue for PROM-based follow-up to capture the dimensions of recovery that matter to patients.

Strengths of this study include the use of repeated PROMIS-PF and PROMIS-APS measures at one, three, six, and twelve weeks after hospital discharge, enabling recovery to be analyzed as a continuous process rather than at isolated timepoints, and the prospective standardized data collection embedded in a randomized controlled trial. However, several limitations should also be acknowledged.

First, PROMIS measures are self-reported and may be influenced by to day-to-day variation. Second, although mixed-effects models capture individual variability, they still suggest one functional shape and may not fully reflect the distinct recovery patterns reported in trajectory- and cluster-based studies. Third, because this analysis was conducted within an RCT, the population may represent a selective subgroup due to the predefined eligibility criteria and because the people who agree to participate in an RCT generally differ from those who decline. In addition, loss to follow-up may have introduced selection bias, as patients without follow-up data were excluded from the analysis. In the parent trial, these patients differed from those included, showing lower baseline functioning, suggesting that patients with poorer preoperative status may be underrepresented in the current analysis. Relatedly, for some preoperative predictors, such as physical inactivity and muscle strengthening activities, there was very limited variability in our sample, which may have reduced the statistical power to detect associations and could partly explain the lack of significant effects for these factors. In addition, risk behavior burden was included as a composite measure representing the cumulative presence of multiple risk behaviors. Although this approach was chosen to capture the overall burden of unhealthy behaviors, observed associations cannot be attributed to any specific risk behavior because the individual behaviors may have contributed differently to recovery outcomes. Furthermore, while the inclusion of patients across multiple surgical specialties enhances the generalizability of the findings to a broad surgical population it also introduces heterogeneity in procedure type and complexity, which may have influenced recovery trajectories and limits the ability to draw conclusions for specific surgical subgroups. Relatedly, surgical oncology was analyzed as a broad dichotomous variable encompassing heterogeneous procedures across multiple surgical specialties. Consequently, the observed associations should be interpreted with caution, as they may reflect differences in surgical complexity, tumor characteristics, treatment pathways, or perioperative care rather than the effect of oncological surgery itself.

Fourth, predictors and their interactions with time were evaluated separately, which assumes independence between predictors. In reality, many factors are correlated, meaning that effects observed in isolation may change when considered together in a multivariable model. Finally, we focused on preoperative characteristics; potentially influential postoperative characteristics or adjuvant treatments (particularly relevant in oncology pathways) were not modeled and could modify recovery.

Given the exploratory nature of this study, our findings are primarily hypothesis-generating and give directions for further research rather than for clinical decision-making. Age and preoperative PROMS emerged as the only characteristics associated with clinically meaningful differences in early recovery, highlighting their potential relevance for managing patient expectations regarding the rate and curvature of postoperative recovery. Other statistically significant factors contributed to between-patient variability in recovery trajectories without exceeding MIC thresholds. While these factors do not support individualized treatment decisions, they may help identify patient subgroups for whom additional pre- or postoperative support may be beneficial. Future research should evaluate these candidate predictors jointly in multivariable prognostic models.

Overall, this exploratory analysis shows that early postoperative functional recovery is characterized by a curvilinear pattern with the most evident improvement occurring early after discharge, and identifies a range of preoperative characteristics associated with postoperative functional recovery trajectories. While age and baseline PROMs showed the differences exceeding the MIC, several other statistically significant factors also contributed to variations in slope and curvature of recovery. Notably, social participation was far less predictable from the same preoperative characteristics than physical functioning, suggesting that additional psychosocial or contextual factors may play a larger role in this domain. Taken together, these findings highlight a set of candidate predictors that warrant further investigation in the development of future prognostic models capable of capturing the multifactorial and dynamic nature of postoperative recovery.

## CRediT authorship contribution statement

**Miriam van der Velde:** Writing – review & editing, Writing – original draft, Supervision, Methodology, Investigation, Data curation, Conceptualization. **Jessica M. de Graaf:** Writing – review & editing, Writing – original draft, Visualization, Methodology, Formal analysis. **Edwin Geleijn:** Writing – review & editing, Methodology, Conceptualization, Funding acquisition. **Karin Valkenet:** Writing – review & editing, Supervision, Methodology, Conceptualization, Funding acquisition. **Marike van der Leeden:** Writing – review & editing, Methodology, Conceptualization, Supervision, Funding acquisition.

## Ethics approval

This study has been approved by the Medical Ethical Committee of Amsterdam University Medical Center, location VUmc, (registration NL61503.029.18), and has been registered at the Overview of Medical Research in the Netherlands (NL-OMON53078).

## Declaration of Generative AI and AI-assisted technologies in the writing process

During the preparation of this work, the authors used Microsoft 365 Copilot and DeepL to refine language and correct typographical or grammatical errors. The authors verified all suggestions, made all substantive decisions, and accept full responsibility for the content of the publication.

## Funding sources

This study received funding from the Taskforce for Applied Research SIA (RAAK.MKB08.004). The funder had no role in the design or conduct of the study; collection, management, analysis, or interpretation of data; preparation, review, or approval of the manuscript; or decision to submit the manuscript for publication.

## Declaration of competing interest

The authors declare that they have no known competing financial interests or personal relationships that could have appeared to influence the work reported in this paper.

## Data Availability

The datasets generated or analyzed during this study are available from the corresponding author on reasonable request.
